# Retraction Note: Association between serum vitamin D levels and the risk of kidney stone: evidence from a meta-analysis

**DOI:** 10.1186/s12937-018-0344-z

**Published:** 2018-03-09

**Authors:** Hai Wang, Libo Man, Guizhong Li, Guanglin Huang, Ning Liu

**Affiliations:** Department of Urology, Beijing Jishuitan Hospital, No. 31, East Xinjiekou Street, Xicheng District, 100035 Beijing, People’s Republic of China

## Retraction

The Editors are retracting this article [1] because post-publication peer review has identified multiple errors in the methodology of this meta-analysis, which invalidate the conclusions drawn. In addition, there is overlap of text with other published articles; the main sources of overlap are [2–5]. The authors do not agree with this retraction.

The online version of this article contains the full text of the retracted article as electronic supplementary material.

## References

[1] Wang H, Man L, Li G, Huang G, Liu N (2016). Association between serum vitamin D levels and the risk of kidney stone: evidence from a meta-analysis. Nutr J.

[2] Zhu Q, Zhang L, Chen X, Zhou J, Liu J, Chen J. Association between zinc level and the risk of preeclampsia: a meta-analysis. Archiv Gynecol Obstet. 2016;293(2):377–82. (First Online 19 September 2015) 10.1007/s00404-015-3883-y 26386964

[3] Ying Z, Jingde C, Qun L, Wei H, Haifeng L, Hong J (2015). Association between breastfeeding and breast cancer risk: evidence from a meta-analysis. Breastfeeding Med.

[4] Tang J, McFann KK, Chonchol MB (2012). Association between serum 25-hydroxyvitamin D and nephrolithiasis: the National Health and nutrition examination survey III, 1988-94. Nephrol Dialysis Transplant.

[5] Leaf DE, Korets R, Taylor EN, Tang J, Asplin JR, Goldfarb DS, Gupta M, Curhan GC (2012). Effect of vitamin D repletion on urinary calcium excretion among kidney stone formers. Clin J Am Soc Nephrol.

